# Transfer Learning-Based Ensemble of CNNs and Vision Transformers for Accurate Melanoma Diagnosis and Image Retrieval

**DOI:** 10.3390/diagnostics15151928

**Published:** 2025-07-31

**Authors:** Murat Sarıateş, Erdal Özbay

**Affiliations:** Department of Computer Engineering, Firat University, Elazig 23119, Türkiye

**Keywords:** melanoma, content-based image retrieval, ensemble, transfer learning, vision transformer

## Abstract

**Background/Objectives**: Melanoma is an aggressive type of skin cancer that poses serious health risks if not detected in its early stages. Although early diagnosis enables effective treatment, delays can result in life-threatening consequences. Traditional diagnostic processes predominantly rely on the subjective expertise of dermatologists, which can lead to variability and time inefficiencies. Consequently, there is an increasing demand for automated systems that can accurately classify melanoma lesions and retrieve visually similar cases to support clinical decision-making. **Methods**: This study proposes a transfer learning (TL)-based deep learning (DL) framework for the classification of melanoma images and the enhancement of content-based image retrieval (CBIR) systems. Pre-trained models including DenseNet121, InceptionV3, Vision Transformer (ViT), and Xception were employed to extract deep feature representations. These features were integrated using a weighted fusion strategy and classified through an Ensemble learning approach designed to capitalize on the complementary strengths of the individual models. The performance of the proposed system was evaluated using classification accuracy and mean Average Precision (mAP) metrics. **Results**: Experimental evaluations demonstrated that the proposed Ensemble model significantly outperformed each standalone model in both classification and retrieval tasks. The Ensemble approach achieved a classification accuracy of **95.25%**. In the CBIR task, the system attained a mean Average Precision (mAP) score of **0.9538**, indicating high retrieval effectiveness. The performance gains were attributed to the synergistic integration of features from diverse model architectures through the ensemble and fusion strategies. **Conclusions**: The findings underscore the effectiveness of TL-based DL models in automating melanoma image classification and enhancing CBIR systems. The integration of deep features from multiple pre-trained models using an Ensemble approach not only improved accuracy but also demonstrated robustness in feature generalization. This approach holds promise for integration into clinical workflows, offering improved diagnostic accuracy and efficiency in the early detection of melanoma.

## 1. Introduction

**Figure 1:**
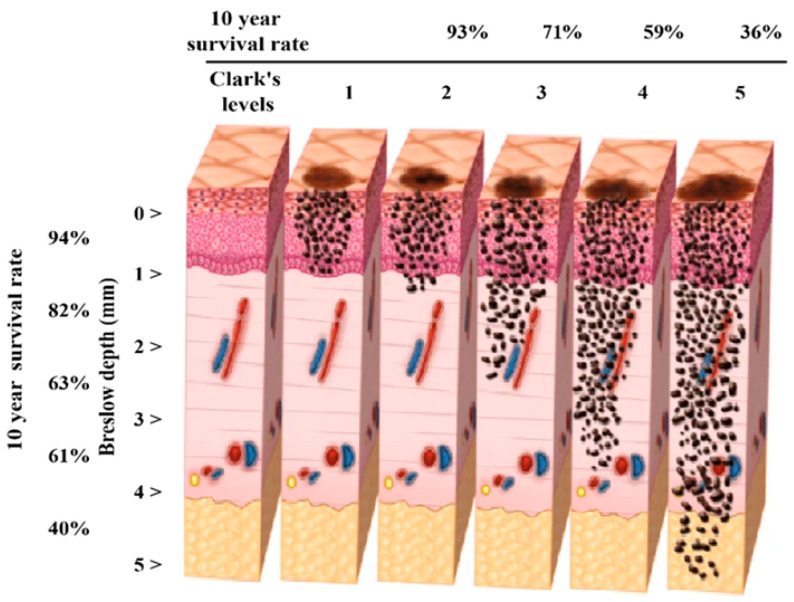
Breslow depth and Clark’s melanoma levels.

Melanoma is among the most lethal and rapidly metastatic types of skin tumors [1]. If not detected early, it can spread swiftly through lymphatic and hematogenous routes. Therefore, early diagnosis is crucial, and in recent years, deep learning-based automatic diagnosis systems have been frequently employed to enhance both the accuracy and speed of diagnostic processes [2]. In particular, transfer learning-based deep architectures enable the development of high-performance classification models even when only limited labeled data are available. One of the most critical parameters in the clinical evaluation of melanoma is staging, with two widely used histopathological systems: Clark level and Breslow thickness [3]. While Clark level indicates the depth of tumor cell invasion into skin layers, Breslow thickness measures the physical distance (in millimeters) from the epidermis’s upper surface to the tumor’s deepest point. These two parameters serve as important prognostic indicators in determining melanoma’s biological behavior and guiding treatment approaches. Indeed, 10-year survival rates can vary significantly—from 93% to 36%—depending on these criteria [4]. Breslow depth and Clark’s melanoma levels are illustrated in Figure 1.

The capability of deep learning systems to provide early warnings by integrating such clinical information offers critical contributions to diagnosis and treatment planning [5]. Consequently, deep learning (DL)-based automatic diagnosis systems have become essential tools to improve the accuracy and speed of diagnostic workflows. Notably, transfer learning-based architectures facilitate the development of robust models even with limited datasets [6,7,8]. Moreover, content-based image retrieval (CBIR) systems provide similarity-based retrieval mechanisms by considering low- and mid-level visual features such as color, texture, shape, and structural patterns. This contributes significantly to decision support systems, especially in discriminating visually complex patterns such as melanoma [9,10].

Early diagnosis of melanoma is vital for patient prognosis and survival. In clinical practice, skin lesion evaluation is commonly performed through dermatoscopic image examination by expert dermatologists. However, this process can negatively impact diagnostic accuracy due to its time-consuming nature and susceptibility to inter-observer variability [11]. Current diagnostic workflows still rely heavily on clinicians’ dermatoscopic examination and lack mechanisms for rapid case retrieval or similar case comparison.

Our proposed dual-task framework addresses this clinical gap by combining high-accuracy classification with content-based image retrieval (CBIR), thus providing both definitive diagnoses and visually similar case references. Although recent CNN- and Vision Transformer (ViT)-based melanoma classifiers report very high accuracy for single tasks, they often overlook two critical requirements: robustness to real-world distribution shifts (e.g., lighting conditions and lesion size variability) and content-based retrieval for visual case comparison. Previous studies have not unified CNNs’ local feature bias and ViTs’ global context modeling within a single ensemble framework that jointly optimizes classification and CBIR performance. Our dual-task ensemble model enhances generalization and delivers interpretable, similarity-based case retrieval.

## 2. Related Works

Despite the growing success of deep learning models in skin lesion classification, most existing studies focus either on CNN-based architectures, which effectively capture local spatial features, or Transformer-based models, which excel at modeling long-range dependencies. However, CNNs often struggle to encode global contextual information, while Vision Transformers (ViTs) typically require large-scale datasets for effective training—often lacking in medical imaging domains. Moreover, most prior research has explored these models in isolation without leveraging their complementary strengths. Very few studies have systematically examined heterogeneous ensembles integrating CNNs and ViTs to improve both diagnostic accuracy and content-based image retrieval (CBIR) performance. This study aims to address this gap by proposing a novel ensemble learning framework combining deep features from DenseNet121, InceptionV3, Xception, and ViT, enabling more robust and generalizable performance in automated melanoma classification and retrieval tasks.

DL techniques, in particular, offer the potential to reduce human dependency in diagnostic processes by making significant advances in medical image processing. Artificial Intelligence (AI), especially DL-based methods, has recently emerged as a promising technology capable of meeting needs in medical image analysis. Transfer learning (TL) techniques, especially, enable highly accurate classification even with limited data. CBIR systems further support the diagnostic process by facilitating rapid retrieval of visually similar examples [12].

AI’s application, particularly DL, has gained substantial attention in skin cancer research. Numerous studies have explored DL algorithms’ potential in accurately classifying and diagnosing various skin cancer types. Simultaneously, research into genetic predispositions, environmental exposures, and lifestyle factors in disease etiology has expanded. The multidisciplinary literature encompasses biomedical engineering, clinical medicine, epidemiology, and computer science, focusing on improving early detection, treatment, and prevention [13].

A prominent line of research integrates human expertise with algorithmic intelligence. For example, a human–computer interaction-based model combining expert clinical judgment with advanced computational methods demonstrated improved diagnostic accuracy and reliability, highlighting the potential of collaborative diagnostic tools leveraging human insight and machine precision [14].

Further advances in medical image analysis were achieved through initiatives like the International Skin Imaging Collaboration (ISIC) and its International Symposium on Biomedical Imaging (ISBI) Challenge, which evaluated novel algorithms for melanoma detection on benchmark datasets, providing critical insights into early diagnosis improvements [15].

Beyond human medicine, DL methods have shown promise in veterinary contexts. One recent study using the MobileNetV2 architecture optimized with RMSprop analyzed images of healthy and diseased cattle skin, achieving ~95% classification accuracy and surpassing existing benchmarks, suggesting DL’s broader diagnostic utility [16].

Recent AI advances have produced increasingly accurate skin cancer classification models. For instance, a hybrid model integrating automated and manual analysis components achieved 93% accuracy and 99.7% recall per class, outperforming both human dermatologists and state-of-the-art machine learning algorithms [17]. Another study proposed a 45-layer deep CNN framework using CLAHE preprocessing to classify skin lesions on the HAM10000 dataset, reaching 99.69% accuracy with low false negatives and high F-measure scores, outperforming previous classifiers in distinguishing melanoma from benign lesions [18].

The SCSO-ResNet50 approach utilized deep feature learning to surpass leading classifiers on benchmark datasets, demonstrating potential for early skin cancer detection with high sensitivity (92.15%) and accuracy (87.72%), aided by multiple feature extraction and dimensionality reduction methods [19]. Similarly, the DCENSnet ensemble combined three DCNNs with Dropout configurations to enhance generalization, achieving 99.53% average accuracy on HAM10000 and excelling across evaluation metrics [20]. The Max Voting ensemble strategy further improved robustness, outperforming five individual ensemble models with a precision rate of 95.80% on the ISIC 2018 and HAM10000 datasets [21].

Recently, hybrid CNN–Transformer models have shown promise in medical image analysis. One study proposed a composite model combining CNN layers with a Vision Transformer (ViT) module to capture local features and global dependencies in dermoscopic images. When evaluated on ISIC 2019, it achieved 92.54% accuracy, outperforming traditional CNN-only models and supporting the addition of Transformer components for enhanced contextual understanding [22]. Another introduced a dual-branch deep learning model integrating ResNet for residual learning and ViT for long-range dependency capture, achieving 95.1% accuracy and 0.971 AUC in melanoma classification, highlighting CNNs’ and Transformers’ complementary roles [23]. Our study extends these directions by employing ViT alongside multiple CNN architectures (DenseNet121, InceptionV3, and Xception) within an ensemble framework, improving classification accuracy to 95.25% and CBIR mean Average Precision (mAP) to 0.9538.

Earlier reviews have highlighted the reliability and efficiency of CNN-based techniques in skin cancer classification, underscoring their importance in future AI-driven dermatological tools [24]. Collectively, these studies emphasize AI’s expanding role in dermatological diagnostics and demonstrate the value of interdisciplinary approaches in advancing the state of the art.

In this study, CBIR mechanisms were employed to support classification performance and contribute to interpretability by facilitating clinician access to similar cases. Notably, the CBIR infrastructure created with ensembled deep feature vectors (feature-level fusion) retrieved clinically meaningful similar cases for each query image with high accuracy. Thus, the system evolved beyond a mere decision-making tool to a multifunctional platform capable of similarity-based case comparison, contextual information presentation, and visual archive exploration.

### The Principal Contributions

Four powerful TL-based architectures (DenseNet121, InceptionV3, Vision Transformer (ViT), and Xception) were used to classify melanoma images. The final layers of these models were rearranged, and unique training protocols were developed for each of them, taking into account variables like memory management, architectural compatibility, and data input size. Patches and PatchEncoder layers were defined specifically for ViT and included in Keras’ special layer registration system (@register_keras_serializable) to ensure that the model can be compiled and loaded correctly. Unlike classical CNNs, this structure processes the visual input by converting it into fixed-size patch arrays and enables the model to learn global context relationships more effectively.

All models were run with dummy input (dummy inference) to ensure that they could be used correctly in the test environment after training and that the graphic structure became active, and the accuracy of the model structures was checked. This process is a mandatory step before the feature extraction process for models with complex input structures, especially ViT and Inception. By using the feature vectors extracted from the intermediate layers of the models before the last layer (especially from fully connected layers such as Dense(512)), the classification process was both supported and integrated into the CBIR system. In this way, each image was represented with a high-dimensional and semantically meaningful vector.

Classification is carried out using a weighted-average ensemble (see Section 3.3), in which each model’s predicted probability is scaled by its respective classification weight.

Retrieval is performed in two phases: first, CBIR is applied individually to each model’s feature vectors; then, feature-level fusion is conducted by scaling each feature vector by its fusion weight and concatenating them into a single ensemble feature representation (see Section 3.5). Classification weights were optimized on validation accuracy/AUC, while fusion weights were tuned on CBIR mAP. Within the scope of the CBIR system, similarity ranking was performed with the cosine similarity metric for 20 random queries using Ensembled feature vectors, and the 5 most similar images for each query were successfully retrieved and visualized. The system, evaluated with Average Precision (AP) and mean Average Precision (mAP) metrics, exhibited high performance with a mAP value of 0.9538. The stability and accuracy of the system were analyzed with precision–recall curves.

A developed structure combines both high-accuracy classification and semantically similar image finding functions in a single system, thus presenting a highly functional and explainable architecture for medical decision support systems. This study presents the foundations of a high-performance visual information retrieval system that can be integrated into both medical image classification and clinical decision support systems, and also demonstrates the applicability of Ensemble architectures in both classification and CBIR areas.

## 3. Materials and Methods

In this study, comprehensive experiments were conducted to compare the performances of DL architectures widely used in the classification of melanoma images and the CBIR field and to analyze the effectiveness of combinatorial approaches [25]. Primarily, DenseNet121, InceptionV3, ViT, and Xception models pre-trained on ImageNet were restructured with the TL method, each integrated with appropriate input sizes and data processing steps [26]. In addition, both individual and Ensemble feature representations were created using the feature extraction capacities of these models, and both classification and CBIR operations were performed on these representations [27]. The Ensemble architecture was structured with the feature-level fusion method to combine the strengths of different models, and model contributions were balanced with the weighted fusion strategy [28]. With this approach, both the generalization ability and similarity-based retrieval performance of the system were increased. Metrics such as accuracy, F1-score, and AUC were used to compare model performances, while AP, mAP, and precision–recall curves were used as a basis for evaluating CBIR performance. All architectures and methods used were tested in a systematic manner, allowing comparative and objective results to be obtained.

### 3.1. Dataset

**Figure 2:**
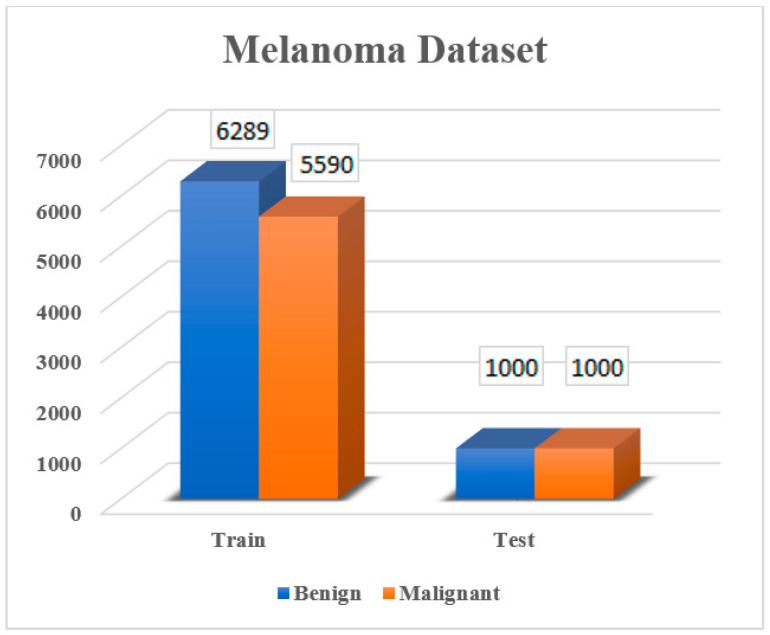
Melanoma dataset image counts.

In this study, the Melanoma Cancer Image dataset obtained from Kaggle, which is equally sized at 224 × 224 pixels, was used to distinguish between benign and malignant lesions [29]. This dataset consists of a total of 13,879 melanoma images, including training (11,879 images) and test (2000 images) data. Figure 2 shows the distribution ratio of benign and malignant images in the dataset.

**Figure 3:**
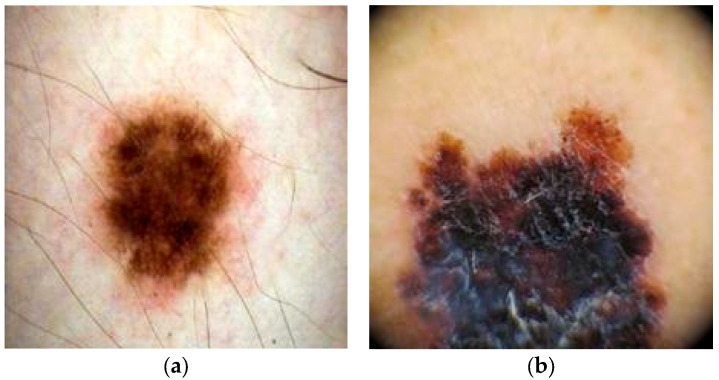
Sample images of (**a**) benign and (**b**) malignant classes from the Melanoma Cancer Image dataset.

Specific to the architecture of each model, the input image dimensions were rescaled. Since the Xception and InceptionV3 models expected an input size of 299 × 299 pixels, all images in the dataset were resized to this size. Before training, we applied real-time data augmentation using Keras’ ImageDataGenerator to improve generalization. Geometric transforms (random rotations up to ±15°, width/height shifts of 10%, and shear up to 0.2) and photometric adjustments (zoom range 0.1 and brightness range [0.8–1.2]) were performed as needed during training. For the DenseNet121 and ViT models, the input size was determined as 224 × 224 pixels. Training and test data were kept separate for all models; training was performed on the training data, while the test data was only used to evaluate the generalization performance of the model. A sample image of benign and malignant classes from the Melanoma Cancer Image dataset is shown in Figure 3.

### 3.2. Transfer Learning (TL)

TL is a method that aims to increase model performance and reduce training time by transferring the knowledge and representation capabilities of DL models previously trained on large-scale datasets to a different but related problem [30]. In this study, TL was utilized in an area where data collection and labeling are difficult in medical image analysis, such as the classification of skin lesions. In particular, it was aimed to accurately classify malignant lesions such as melanoma using deep Convolutional Neural Networks (CNNs) pre-trained on natural images.

**Figure 4:**
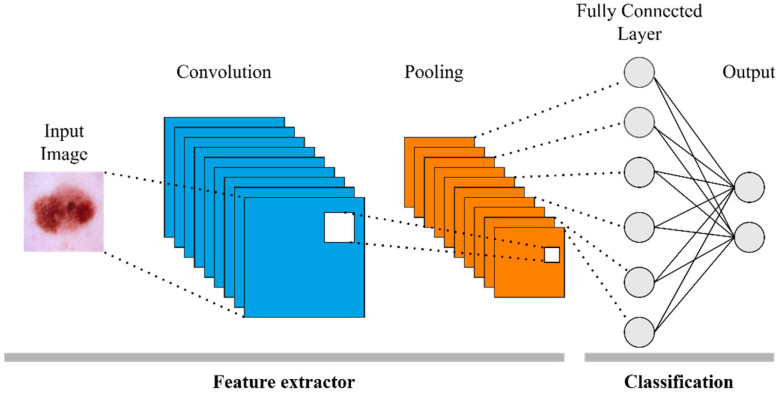
Basic CNN architecture.

Figure 4 shows that the skin lesion image given as the input of the basic CNN architecture is transformed into meaningful feature maps by passing through successive convolution and pooling layers [31]. These features are processed with the subsequent fully connected layers to reach the classification result.

This structure is generally used in the TL context by reusing the “feature extraction” part of a pre-trained model and retraining only the “classification” part. In this part of the study, TL was implemented using powerful and up-to-date DL architectures such as DenseNet121, InceptionV3, ViT, and Xception in order to achieve higher accuracy in the melanoma classification task. After evaluating the individual classification performances of each model, fine-tuning operations were performed on the TL InceptionV3 model, and the classification performance was increased. Then, the outputs of these 4 models were combined with the Ensemble learning method to try to achieve higher overall success.

First, the DenseNet121 model was loaded using the weights (weights = “ImageNet”) previously trained on the ImageNet dataset. With the include_top = False statement, the fully connected layers of the model were excluded, and only the feature extractor part was used. Training for the first 121 layers of the model was closed by setting trainable = False, thus preserving the previously learned general features (basic information such as edge, texture, and color) and preventing the weights from being changed during training. We froze the first 121 layers to preserve these generic ImageNet-learned filters, while fine-tuning only the upper blocks to adapt specifically to melanoma features. This step is the main advantage of transfer learning, which significantly reduces the training time and provides higher generalization performance with limited data. After the output of the DenseNet121 base model, the following layers were added sequentially:

- Conv2D and MaxPooling Layers: Three Conv2D layers contain 256, 256, and 128 filters, respectively. These layers are added to provide high-level feature extraction, and each of them is activated with the ReLU activation function. The ReLU function increases computational efficiency while facilitating the model in learning nonlinear relationships. MaxPooling2D layers following each Conv2D layer are added to reduce spatial dimensions and noise in the data.
- GlobalAveragePooling2D and Flatten Layer: With the GlobalAveragePooling2D layer, the average value of each filter channel is taken, and the multidimensional data is reduced to a summarizing format. The Flatten layer transforms this data into a one-dimensional vector to be presented as input to the subsequent Dense layers.
- Dense and Dropout Layers: High-dimensional features obtained with the Dense layer (512 neurons; ReLU activation) have been made more abstract and distinctive. Dropout (50%) has been added to prevent overfitting and increase the generalization ability of the model.
- Output Layer: Since the dataset has two classes, the output layer has 1 neuron and a sigmoid activation function.

After the layers of the model were designed, the optimization algorithm and training parameters affecting the training process were defined. With ModelCheckpoint, the weights with the highest validation performance were recorded, and with EarlyStopping (patience = 10 epochs), the training process was automatically terminated if the validation accuracy did not improve for a certain period of time. Thus, unnecessary epochs were prevented and the training process was optimized. Adamax was selected as the optimization algorithm, and the learning rate was determined as 0.001. The batch size was selected as 32, and the maximum epoch number was set as 40. Values such as learning rate and max epoch were selected to be the same for all other models.

Secondly, the Xception model was loaded using the weights trained on the ImageNet dataset and used as a feature extractor by excluding the classifier layers (include_top = False). The first 80 layers of the model were frozen by closing the training. Freezing the first 80 layers retains generic filters, allowing the remaining layers to specialize into melanoma patterns. Three Conv2D layers (256, 256, and 128 filters; ReLU activation) and the subsequent MaxPooling2D layers were added to the output of the model. Finally, overlearning was prevented by using GlobalAveragePooling2D, Flatten, Dense (512 neurons; ReLU activation), and 50% Dropout layers. Melanoma classification was performed using a single neuron with a sigmoid function in the output layer. During the training process, the Adamax optimization algorithm (learning rate = 0.001), EarlyStopping, and ModelCheckpoint methods were used to maximize model performance.

**Figure 5:**
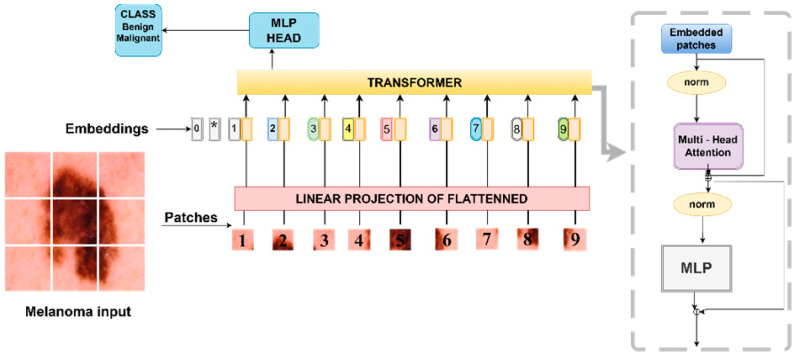
The flowchart of the ViT architecture.

Thirdly, unlike the traditional CNN structure, the ViT model processes the input images by dividing them into patches and processing them through Transformer blocks [32]. In this study, the images were divided into 16 × 16 patches (patch_size = 16), projected to 64-dimensional embeddings (projection_dim = 64), and processed through multi-head self-attention with 4 heads (num_heads = 4) and 8 Transformer blocks (transformer_layers = 8). The resulting features were then passed through an MLP head with layers of 2048 and 1024 units (mlp_head_units = [2048, 1024]), with Dropout applied to prevent overlearning [33]. Binary classification was performed via a sigmoid output that was trained using Adam (learning_rate = 0.001) and evaluated using a classification report, ROC curve, and confusion matrix. The ViT architecture is illustrated in Figure 5.

Finally, the InceptionV3 model is loaded with weights trained on ImageNet, and all layers are initially frozen (trainable = False). Four Conv2D (512, 512, 256, and 128 filters; ReLU activation) and MaxPooling2D layers are added to the model, and then the data is made one-dimensional with BatchNormalization and GlobalAveragePooling2D layers. Then, a densely connected Dense layer (512 neurons; ReLU) and Dropout (50%) are added. Finally, a sigmoid-activated output layer is used. In this first training phase, the classification performance of the model is optimized using the Adamax optimization algorithm (learning rate = 0.001), EarlyStopping, ReduceLROnPlateau, and ModelCheckpoint methods. In this direction, 89.7% test accuracy is obtained.

In order for the InceptionV3 model to achieve higher accuracy, the first 150 layers of the previously frozen InceptionV3 base model were kept fixed, allowing higher-level layers to be opened for training. Empirically, unfreezing only the last ~50 blocks (layers 151–mixed10) yielded the best trade-off between feature reuse and melanoma-specific specialization. In this way, the model was aimed to learn high-level features specific to the current task of melanoma classification. During the fine-tuning process, a lower learning rate (learning rate = 0.0001) was used to ensure that the model learned the dataset-specific features better. During training, the model was further improved with methods such as EarlyStopping, ReduceLROnPlateau, and ModelCheckpoint. As a result of fine-tuning, the classification performance of the InceptionV3 model on the test dataset was calculated as 91.2%, and the overall success level was increased by 1.5%.

### 3.3. Ensemble Learning (EL)

**Figure 6:**
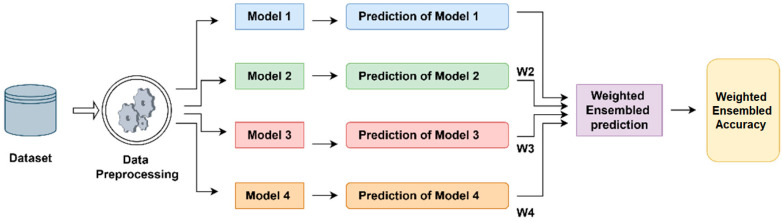
The flowchart of the weighted Ensemble average technique.

Weighted Ensemble learning is an approach that aims to improve the overall classification performance by combining different outputs of multiple models in certain ratios (weights). Here, the predictions of each model are multiplied by weights that are predetermined or optimized on the validation set, and the resulting values are added to produce the final probability. Then, the classification decision is made according to this probability. This technique can guarantee that faults in one model are offset by those in other models by combining the advantages of many architectures [34]. Weight selection can be achieved with fixed rules, such as simply giving equal weights, or with systematic methods such as Random search, Grid search, or Bayesian optimization [35]. An example flowchart of the weighted Ensemble average technique is shown in Figure 6.

While developing the ensemble model, the registered DenseNet121, InceptionV3, ViT, and Xception models that were pre-trained with the TL approach were initially called. In particular, special layer definitions (patches and PatchEncoder) used in the ViT model were registered with tf.keras.utils.register_keras_serializable() and integrated into the model. Thus, each model can be automatically recreated during the testing phase [36].

The final estimate is obtained by combining the prediction probabilities (model outputs) given by four different models with predetermined weights within the framework of W_DenseNet_, W_Inception_, W_ViT_, and W_Xception_. At the formula level, the final probability value for each sample is given in Equation (1).

(1) $$ŷ=\frac{w_{1}\;p_{1\;\;}+w_{2}\;p_{2\;}+\cdots +w_{n}\;p_{n\;\;}}{w_{1}+w_{2}+\cdots +w_{n}}$$

**Figure 7:**
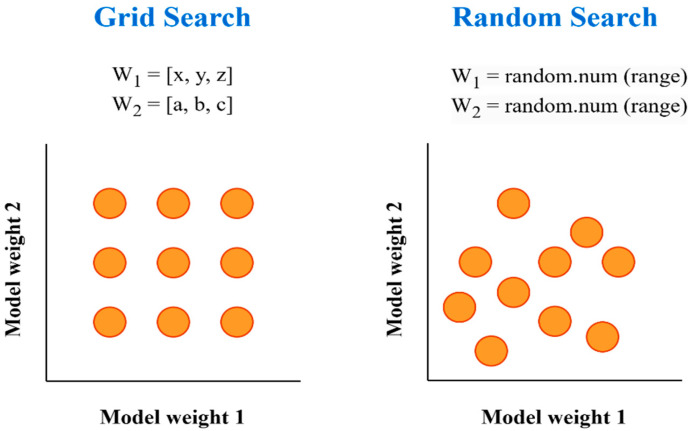
The screening strategies of Grid search and Random search methods in the hyperparameter domain technique.

In this study, the probability equation (threshold) is determined as 0.5. If the weighted Ensemble result is ŷ ≥ 0.5, class = 1; if ŷ ≤ 0.5, class = 0. Initially, the weights to be given to each model were assigned according to the individual accuracy performance of the model; that is, more weight was assigned to the models that gave better results in the validation. Then, beyond this preliminary assignment, the weights were fine-tuned using the Random search method. We sampled each model’s weight from Uniform (0, 1) and normalized it so that ∑w_i_ = 1, then ran up to 2000 random trials—terminating early after 100 consecutive non-improving draws on validation AUC—and chose the weight set that maximized AUC. Figure 7 shows how the Grid search and Random search methods, used to try different combinations of weights, work in the hyperparameter space.

Random search is an effective approach used for hyperparameter optimization. With this method, randomly selected parameter values (weights of each model in this study) within a certain range were optimized on the validation set. In each trial, the obtained Ensemble model was evaluated on the training and test datasets. Then, the parameter combinations that improved these metrics were recorded, and the parameter set (i.e., weights) that reached the highest accuracy value was selected to obtain the final Ensemble averaging model. The final weights obtained after the Random search provided a significant improvement in reaching the targeted accuracy. Thus, it was observed that the Ensemble learning approach, in which different architectures were combined, increased the overall accuracy and other performance metrics in melanoma classification problems. The classification accuracy of the developed Ensemble model on the test dataset was obtained as 95.25%, and other performance metrics were also recorded.

### 3.4. Content-Based Image Retrieval (CBIR)

**Figure 8:**
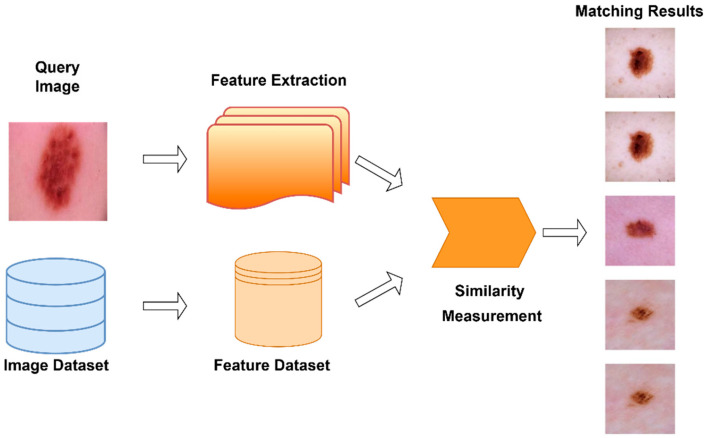
The implementation of the CBIR technique for the detection of melanoma.

CBIR is an information retrieval system that automatically retrieves similar images based on the content of images [37]. In this approach, images are represented not only with labels or text-based descriptions, but directly using visual features (color, texture, shape, structural patterns, etc.). In this way, images with similar content to a query image are obtained from the database by ranking them according to numerical similarity criteria. Much more successful results are obtained with high-dimensional deep feature vectors obtained with deep learning-based methods [38]. Architectures with strong representation learning capabilities significantly increase CBIR performance. Figure 8 visualizes the CBIR technique performed for queries and matches performed for melanoma detection in this study.

CBIR systems are effectively used in many critical applications such as retrieving similar cases in the field of medical image analysis, strengthening diagnostic support systems, creating educational materials, and accelerating clinical decision processes.

In this study, CBIR systems were developed using both individual models and the Ensemble model after TL models and the Ensemble model classification operations were performed on melanoma images; the aim of these systems was to recall similar medical images with feature-fusion-level operations. In order to visualize the performance of the system, the 5 most similar images were determined for one of the 20 randomly selected query images from the dataset, and the cosine similarity scores between these images and the query were calculated. In addition, the precision–recall (PR) curve for this query was drawn, and the success of the CBIR system in retrieving the relevant images was evaluated visually. The overall performance of the system was evaluated with the mAP value and cosine similarity metrics calculated for 20 queries.

While calculating the cosine similarity in the developed CBIR models, each image is first represented with a numerical vector in order to compare the images. This vector numerically summarizes the content of the image (color, shape, texture, semantic information, etc.). Therefore, cosine similarity is a metric that measures the similarity between two vectors based on the angle between them. It is frequently preferred in CBIR systems that work with high-dimensional and deep learning-based feature vectors. This method takes into account the orientation of the vectors rather than their magnitude, so it is more robust to lighting differences or scale changes. The similarity between two vectors is calculated by cosine similarity as given in Equation (2):

(2) $$cosine_{similarity\left(x,y\right)}=\frac{x\cdot y}{\left\|x\|\cdot \|y\right\|}$$

The general steps of developed CBIR systems are listed below:

- Extracting features from images;
- Storing feature vectors in a database;
- Extracting features from the query image;
- Calculating distance/similarity between the query vector and database vectors;
- Finally, returning the images with the lowest distance or highest similarity.

In the first stage of the study, the previously trained and saved DenseNet121 model was loaded, and then the appropriate layer was selected for feature extraction from this model. Especially for the DenseNet121 architecture, global average pooling layers such as avg_pool or global_average_pooling2d were generally preferred to obtain compact and meaningful feature representations. With the defined vector feature function, each image is converted into a high-dimensional feature vector by passing it through the appropriate layer in the DenseNet121 model. The feature vectors of all images in the dataset were pre-calculated and stored as numpy arrays. Thus, there was no need for repeated calculations during the query phase, and the processing speed was increased. All images were fed to the DenseNet121 model to obtain feature vectors, and these feature vectors and the labels of the relevant images were saved in files (in .npy format). The similarity between images was calculated with the cosine similarity metric between the angles of the feature vectors. Feature extraction was applied to the images in the entire dataset, and the performance data obtained as a result of the similarity search performed on 20 randomly selected query images from the test dataset was evaluated with the help of mAP, AP, and cosine similarity values. In addition, a PR curve was created for the sample query in order to visually examine the success of the system in retrieving the relevant images. In the experimental study, the developed CBIR system achieved a high average sensitivity mAP value of 0.9496 and achieved the success of retrieving the relevant images with great accuracy.

In the InceptionV3 model, an architecture that can capture details of different dimensions was preferred, thanks to the Inception blocks where multi-scale convolutional filters are used. The model was finalized with pre-trained weights and an additional global average pooling layer and fine-tuned. The images in the entire dataset were passed through the intermediate layer of this fine-tuned model to create high-dimensional feature vectors. After storing these vectors, feature extraction was performed for the query images in a similar manner, and similarity rankings were obtained by calculating cosine similarity with the vectors in the database. The Xception architecture aimed to reduce complexity relatively and gain high representation power by using the discrete convolution method per depth. The pre-trained Xception model was adapted to the CBIR purpose with the vector output obtained from the intermediate layers instead of the final layers. All images in the dataset were passed through the model, and feature vectors were extracted and stored in .npy format. In the query phase, the same feature extraction step was applied to randomly selected images from the test dataset; then, similarity analyses (cosine similarity, AP, mAP, and PR curve) were performed to evaluate the model’s retrieval success numerically and visually. In the ViT-based approach, images were divided into specific patches and processed with the multi-head attention mechanism of a Transformer architecture. This model produces rich feature vectors that include the global context by considering the embedding representations produced for each patch. As in other architectures, the intermediate output from ViT’s pooling method is the basic vector representation used for CBIR. After these vectors were produced and recorded for all images in the database, the feature vector obtained during the query was compared with those in the database to create similarity scores (cosine similarity). The AP and mAP values of the randomly selected queries were examined, and PR curves were also obtained.

### 3.5. Multi-Model (Feature-Level Fusion)

At this stage, after examining the results obtained with individual models (DenseNet121, InceptionV3, ViT, and Xception), we aimed to reach higher CBIR performance by combining the strengths of these models with the feature-level fusion approach [39,40,41]. First, separate ImageDataGenerator and flow_from_directory operations are defined for models accepting inputs of different sizes (224 × 224 or 299 × 299), so that each model receives training images in the size it expects. DenseNet121, InceptionV3, ViT, and Xception models are loaded from the colab environment registered with tf.keras.models.load_model. A “feature extraction model” is created from each model to receive the output of an intermediate layer before the last layers, and a function called get_feature_model is defined for this process. Thus, instead of the main classification layer, the output of the layer where the model produces a rich feature representation is accessed. The dataset is fed to the relevant model in an appropriate size, and a feature vector is obtained for each image. With dense_features, xception_features, vit_features, and inception_features, each model’s feature is a matrix (*N* × *d*) with as many rows as the number of images in the test dataset (*N*) and columns (*d*) as the model’s last layer output size. In the concatenation section, all model outputs were combined horizontally (np.concatenate(…, axis = 1)); that is, feature vectors were added side by side in a single row to form an “ensemble” feature vector. Thus, each image is now represented by a single vector (row) with a longer dimension carrying the information of four models. The critical point of the feature-level fusion approach is to transform the feature vectors produced by each model into a single large vector by the “horizontal concatenation” method.

After the completion of the concatenation process, the CBIR performance is measured using the Ensemble feature vectors obtained. In particular, 20 queries were randomly selected from the test dataset, and the vector created for each query was compared with the Ensemble vectors of all images of the dataset (gallery). Cosine similarity (cosine_similarity) was used as the comparison method; thus, the closest (similar) vectors to the query were determined. After the similarity scores were sorted from largest to smallest, the AP value was calculated depending on the positions of the images belonging to the same class/label as the query in the score order. mAP was obtained by taking the average of the AP values of these 20 randomly selected queries. Precision and recall values were extracted for each query with the precision_recall_curve function and shown on the graph, and all the curves drawn separately for the 20 queries were combined in a graph, or an average PR curve was created. Experimental results show that the developed multi-model CBIR approach achieves a mAP value of 0.9538, outperforming the scenarios using each model alone.

## 4. Experimental Results and Discussion

In this study, a dataset consisting of 13,879 melanoma images (11,879 training and 2000 test data points) was used. In the first stage, DenseNet121, InceptionV3, ViT, and Xception models were trained separately, and the classification performance of each was evaluated in detail (on the test dataset). Then, different weights were assigned based on the classification accuracy rates of these models, and the averaging Ensemble learning method was applied. Thus, an Ensemble model expected to combine the strengths of different architectures was obtained, and the results were compared. In the next stage, CBIR operation was performed independently with each model, and in the last stage, it was aimed to further improve the CBIR performance by using models combined with the feature-level fusion approach. In this context, both the classification and CBIR performances of the four models in question were compared, and it was shown that higher classification and CBIR results could be achieved by combining the strengths obtained through the Ensemble methods.

### 4.1. Performance Metrics

Accuracy measurements from model performance evaluation metrics were used to compare results and make better choices. Computation accuracy, precision, recall, F1-score, and the ROC curve are important metrics used in the evaluation of classification models, particularly in ML and DL. These metrics help assess the performance of a model in predicting outcomes. They are calculated based on four fundamental values: true positives (*TP*), true negatives (*TN*), false positives (*FP*), and false negatives (*FN*) [42]. The confusion matrix presented in Table 1’s metrics assesses the classification performance. In addition, the CBIR performance of the models was measured separately using feature vectors. Accuracy measurements from model performance evaluation metrics were used to compare results and make better choices. Computation accuracy, precision, recall, F1-score, and the ROC curve are important in Ensemble learning. The CBIR performance of the Ensemble model was evaluated with the developed multi-model approach. Extensive experiments were conducted to examine the effects of the used parameters and the developed models on classification and CBIR performance, and the obtained results are presented comparatively.

The overall accuracy of the model’s predictions is measured by accuracy. This is mathematically calculated in Equation (3):

(3) $$Accuracy=\frac{\left(TP+TN\right)}{\left(TP+TN+FP+FN\right)}$$

The precision of the model is determined by dividing all of its positive predictions by the percentage of true positive forecasts. It indicates the model’s ability to avoid false positives. This is mathematically calculated in Equation (4):

(4) $$Precision=\frac{TP}{\left(TP+FP\right)}$$

The percentage of accurate positive predictions among all real positive events in the dataset is measured by recall. It indicates the model’s ability to capture all positive instances. This is mathematically calculated in Equation (5):

(5) $$Recall=\frac{TP}{\left(TP+FN\right)}$$

The harmonic mean of recall and precision is known as the F1-score. It offers a measure that strikes a compromise between recall and precision by taking false negatives and positives into account. This is mathematically calculated in Equation (6):

(6) $$F1\;score=2*\frac{\left(precision*recall\right)}{\left(precision+recall\right)}$$

The Area Under the Curve (AUC) and Receiver Operating Characteristic (ROC) curve are graphical tools for assessing how well categorization algorithms work. The area under the ROC curve, or AUC, indicates how well a model performs when it is closer to 1. Comprehension of a classification model’s performance requires a comprehension of these metrics. While accuracy gives a general idea of the model’s correctness, precision, recall, and F1-score provide information about how well the model can capture all positive instances, produce accurate positive predictions, and balance precision and recall.

The metrics used for the performance evaluation of the CBIR method are explained below:

PR Curve: This is a graphical method widely used to measure the performance of CBIR systems. It shows the relationship between precision and recall values. This curve expresses how accurately the system retrieves the relevant images in the dataset (precision) and how many of all relevant images it can retrieve (recall).

AP: This summarizes the relationship between precision and recall values in the ranking of the retrieval results obtained for a specific query image as a single numerical value. A high AP value indicates that the system can successfully rank the relevant images.

mAP: This is the average of AP values calculated for multiple query images. This value is a single metric that summarizes the performance of the overall CBIR system. A high mAP value indicates that the CBIR system consistently provides successful results.

Cosine Similarity: This is a mathematical metric used to measure the similarity of feature vectors generated between two images. Cosine similarity takes values between 0 and 1. As the value approaches 1, this means that the features of the two images are similar, and therefore, the system successfully retrieves similar images. CBIR performance evaluation metrics play an important role in presenting the effectiveness of the models and the success rate of the developed feature-level fusion method in a detailed and comparative manner.

### 4.2. Performance Evaluations

In the performance evaluation of the developed melanoma classification method, the effectiveness was determined by tests performed on the models used. In this respect, the performance evaluation was made based on the accuracy rates of the models. In addition, the precision, recall, and F-score metrics of the models were calculated using the complexity matrix for a more detailed performance evaluation.

The training process of all models was carried out using fixed hyperparameters in order to obtain comparable results. In this context, 32 was selected as the batch_size, 40 epochs for the training period, and 0.001 for the learning rate. The training process using fixed hyperparameters provided the opportunity to compare the performance of different architectures objectively, while allowing the obtained results to reveal the differences specific to the model architecture more clearly.

**Figure 9:**
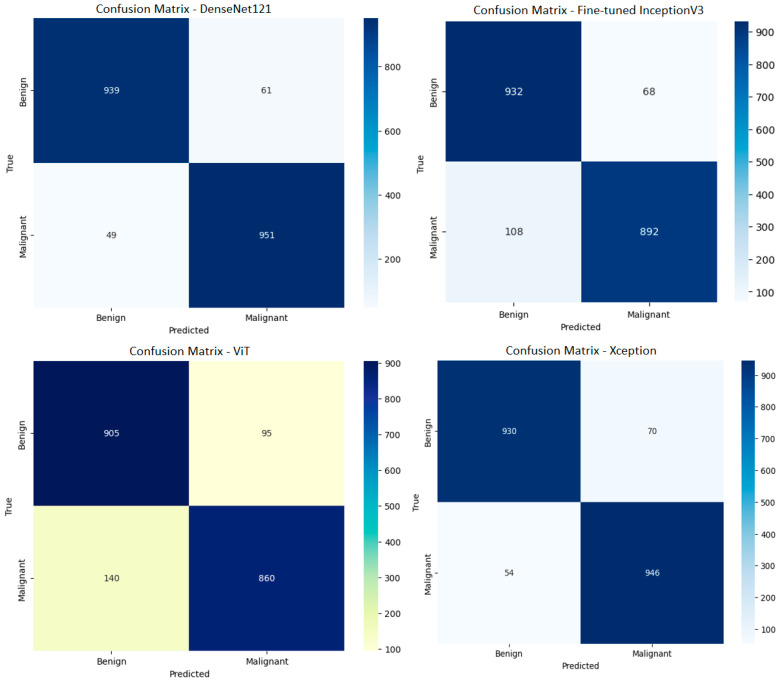
Confusion matrices of the DenseNet121, fine-tuned InceptionV3, ViT, and Xception models.

**Figure 10:**
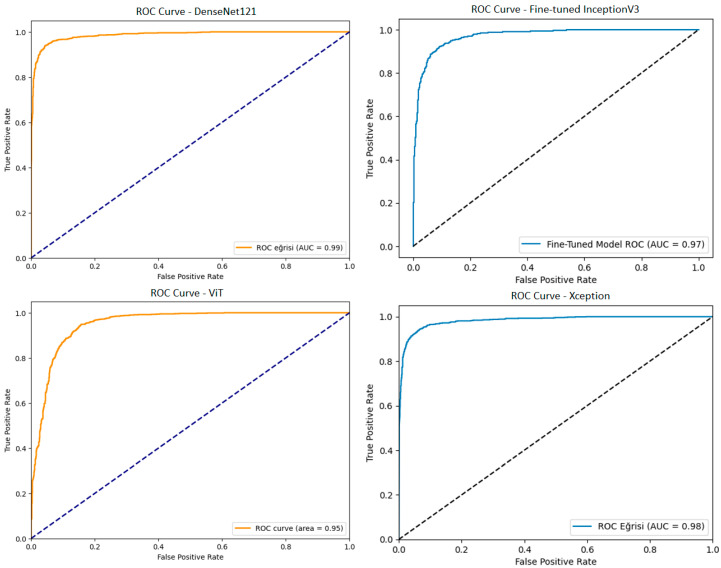
ROC curves of the DenseNet121, fine-tuned InceptionV3, ViT, and Xception models.

First of all, the necessary optimizations were made with the TL method, and the classification processes were performed with the DenseNet121, InceptionV3, ViT, and Xception architectures. The confusion matrices of the performance obtained by these 4 models are shown in Figure 9. The ROC curves of these models are shown in Figure 10. The results in terms of different performance metrics obtained through confusion matrices are given in Table 2.

As given in Table 2, all performance metrics obtained from the confusion matrices of the four models are evaluated holistically. In addition to providing high-performance metrics such as 94.50% accuracy, 93.90% precision, 95.04% recall, and 94.47% F1-score, the DenseNet121 model also demonstrated its classification power with an AUC value of 0.99 obtained in the ROC curve analysis. Low misclassification rates in the confusion matrix indicate almost complete discrimination of both benign and malignant samples. The InceptionV3 model has 91.20% accuracy, 93.20% precision, 89.62% recall, and 91.37% F1-score values, and the ROC curve analysis reveals that the model exhibits sufficient discrimination with an AUC of 0.97; however, as seen in the confusion matrix, more false negatives (*FN*) were observed, especially for the malignant class. The Xception model offers a balanced performance with 93.80% accuracy, 93% precision, 94.51% recall, and 93.75% F1-score, and shows high discrimination in the ROC curve with an AUC value of 0.98. The distribution in the confusion matrix shows that Xception has a low error rate in distinguishing benign and malignant samples. The ViT model, on the other hand, exhibits lower performance compared to other models with 88.25% accuracy, 90.50% precision, 86.60% recall, and 88.50% F1-score; while the ROC curve analysis gives an AUC value of 0.95, the higher false negative rate observed in the confusion matrix suggests that the model may be inadequate in the detection of malignant cases, which are of critical importance especially in clinical applications. In light of these data, performance metrics supported by both ROC curve and confusion matrix analyses show that the DenseNet121 and Xception models provide superior and clinically reliable results for melanoma detection. Especially in applications where the false negative rate is critical, the high sensitivity and specificity values of these models provide advantages for their potential clinical integration.

**Figure 11:**
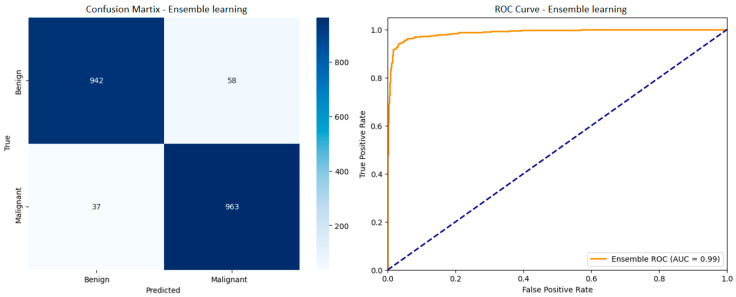
Confusion matrix and ROC curve of the Ensemble learning model.

After the classification operations were performed with 4 TL models, the aim was to create a single combined model by assigning different weights to each model based on the classification performances of individual models with the averaging Ensemble learning method. This approach aims to reduce the impact of the weaknesses of individual models while highlighting their strengths by blending the performance metrics obtained by architectures such as DenseNet121, InceptionV3, ViT, and Xception separately. Thus, the Ensemble model has the potential to offer higher overall accuracy and consistency by minimizing the classification errors observed in most of the models used alone. The confusion matrix and ROC curve of the Ensemble learning model are shown in Figure 11, and the performance metrics are given in Table 3.

The Ensemble learning approach shows a significant improvement when compared to the performance metrics of individual models. Among the individual models, the highest accuracy is 94.50% with DenseNet121, and the highest recall is around 95% in DenseNet121 and Xception models, while the Ensemble model stands out with 95.25% accuracy and 96.22% recall values. In addition, the 94.20% precision and 95.20% F1-score values of the Ensemble model generally exceed the metrics of all individual models, thus minimizing the classification errors and providing a more balanced and reliable distinction between benign and malignant samples. These findings reveal the potential of the Ensemble learning strategy to reduce false negative rates and increase the overall classification success, which are critical in clinical applications, by compensating for individual model errors.

In this study, in order to examine the benefits of the CBIR technique in medical image analysis, the comparative CBIR performances of four different TL-based architectures, namely DenseNet121, InceptionV3, ViT, and Xception, were evaluated for melanoma detection. In the CBIR operations performed on the feature vectors obtained by the models, 20 queries were randomly selected for each model and evaluated with different metrics such as PR curves, mAP, AP, five similar images, and cosine similarity scores of the images. Thus, the ability of each architecture to detect similar images and the potential to minimize error rates in clinical applications were revealed. This approach evaluates the similarity-based search performances of different DL architectures from a holistic perspective and provides important findings for determining the most appropriate model in medical image analysis.

**Figure 12:**
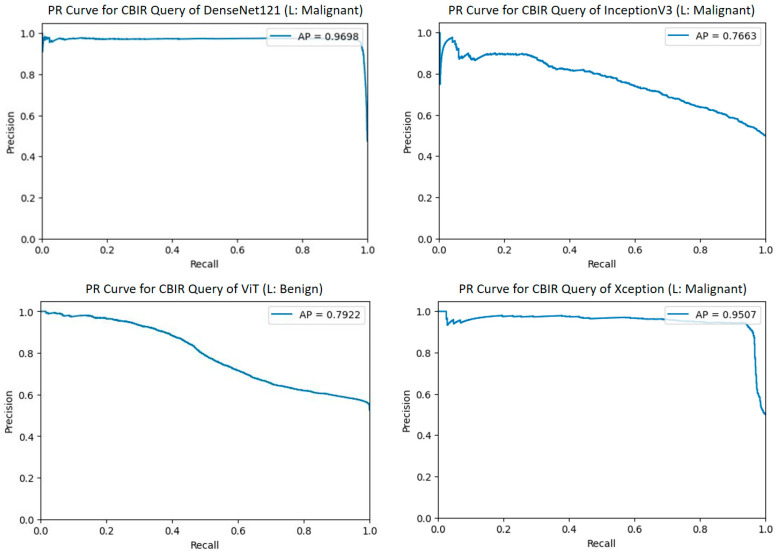
PR curves and AP values for CBIR performance of the Transfer learning models.

**Figure 13:**
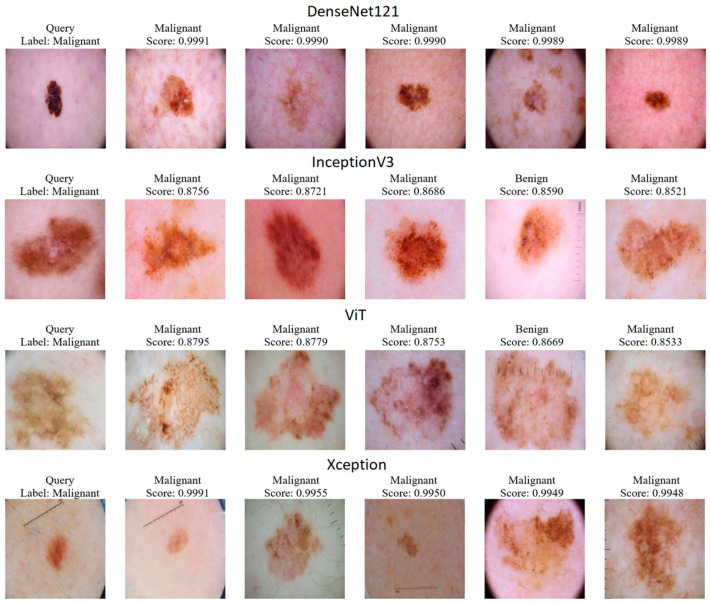
Five similar image results on CBIR performance of Transfer learning model queries.

Figure 12 shows the PR curves and AP values given for one of twenty random queries for the CBIR performance of DenseNet121, InceptionV3, ViT, and Xception models. Figure 13 shows five similar images and the resulting query scores. The CBIR performance results of the models for the mAP values of 20 queries are given in Table 4.

When examining Figure 13, DenseNet121 and Xception models generally retrieve visually similar lesions correctly, while InceptionV3 and ViT produced erroneous matches in some cases. For example, InceptionV3, in the fourth image (a lesion with an irregular, low-contrast border and subtle color variegation), prioritized global color histograms and overlooked fine border details, matching it to a benign example. ViT, with its patch-based attention mechanism, overfocused on specular highlights and glare artifacts in the same image; these high-frequency patterns overshadowed the true tissue texture. Both models also confused highly heterogeneous pigmented lesions—characterized by a “speckled” appearance—with benign samples, generating incorrect similarities.

These findings suggest two avenues for improvement:

- Preprocessing to remove hair and glare artifacts, for example, using inpainting or DullRazor.
- Employing boundary-aware descriptors (e.g., explicit contour or texture filters) that favor irregular edges over bulk color statistics.

This analysis aims to expose the models’ limitations and guide future work toward more robust CBIR approaches.

**Table 4:** CBIR performance results of the models for the mAP values of 20 queries.

Models	Mean Average Precision (mAP)
DenseNet121	0.9496
InceptionV3	0.7922
ViT	0.7539
Xception	0.9171

DenseNet121, with its high AP value of 0.9698 obtained in the sample query and its mAP value of 0.9496 calculated over 20 queries, shows that it produces detailed and deep feature maps that represent the significant malignant features in the query image with high accuracy. The high initial precision value in the PR curve and the consistent performance in the wide recall range indicate that DenseNet121 clearly distinguishes between classes by minimizing the FP rate.

The Xception model also offers a similarly strong performance; the mAP value of 0.9171 shows that the model can efficiently separate features thanks to the depth-separated convolutional layers. The PR curve shows a slight decrease as recall increases while maintaining a high precision value; this shows that the model ranks the correct malignant examples in the top ranks during retrieval.

InceptionV3, with its mAP value of 0.7922, exhibits a significant performance decrease compared to other models. The PR curve shows a sharp decrease in precision in high-recall regions. This situation showed that the model may be insufficient in distinguishing benign and malignant features, and proportionally, the model returned one benign image for the malignant query during retrieval. This situation technically expresses that the distinction between the two classes in the model’s feature space is more blurred.

ViT offers the lowest performance with 0.7539 mAP. Transformer architecture generally requires a larger dataset. In PR curves, the significant decrease in precision, especially with the increase in recall, shows that ViT cannot demonstrate the expected discriminatory power in feature representation.

In summary, DenseNet121 and Xception exhibit reliable performance in clinically critical retrieval tasks by producing detailed and discriminatory features for sample malignant query images with high mAP values and stable PR curves. On the other hand, InceptionV3 and ViT show insufficient feature discrimination, especially with decreases in precision in high-recall regions and the presence of benign samples in the retrieval results despite the sample malignant query. These technical data highlight the importance of choosing models that provide high discrimination and low false positive rates in CBIR applications.

**Figure 14:**
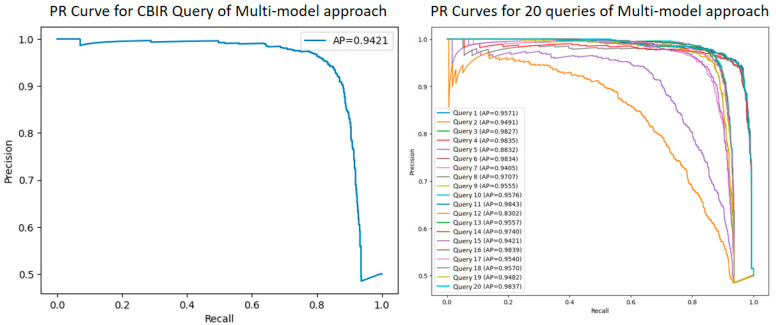
PR curves and AP values for CBIR performance of the multi-model approach.

**Figure 15:**
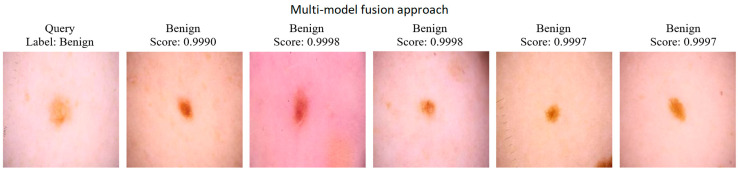
Five similar image results on the CBIR performance of the multi-model approach.

After evaluating the CBIR performance of individual models, the proposed feature-level fusion approach was applied to combine the strengths of DenseNet121, InceptionV3, ViT, and Xception models. Within the scope of this method, an enriched Ensemble feature vector was created by combining the feature vectors obtained from each model horizontally. The CBIR performance of the multi-model fusion approach was evaluated on 20 randomly selected query images, and mAP values were measured using cosine similarity in the query–gallery similarity calculation. Figure 14 shows the PR curves and AP values given for one and then all twenty random queries for the CBIR performance of the multi-model fusion approach. Figure 15 shows 5 similar image retrievals and scores for the CBIR performance of the multi-model fusion approach. In addition, the mAP values of the 20 queries for the CBIR performance of the multi-model fusion approach are given in Table 5.

With the developed multi-model (feature-level) fusion, a rich and detailed representation was provided in the CBIR task by using the strengths of four different architectures, such as DenseNet121, InceptionV3, ViT, and Xception. The specially defined get_feature_model function was used to obtain the outputs of individual models in the intermediate layers before the last classification layer. In this way, the deep feature vectors produced by each model could be used as discriminative representations of objects instead of the classification process. With the feature-level fusion method, the feature vectors obtained from each model were directly combined (concatenation) horizontally. The technical advantages of this approach are as follows:

- Each model can capture different features of the image thanks to different architectural structures and learning capacities. While DenseNet121 and Xception provide high-discrimination features, ViT and InceptionV3 can provide different information at certain scales or textures. This complementary structure enables a broad perspective that a single model cannot capture.
- Combining features obtained from different architectures allowed the model to learn both general and specific discriminative features. This allowed the system to produce consistent and high-performance results against different types of queries.
- In the high-dimensional feature space formed after the concatenation process, similarity measurements (cosine similarity) naturally highlighted the effect of more discriminative components. Thus, the features produced by models with higher performance indirectly became dominant in the collective representation.

The obtained results clearly show how strong the CBIR performance of the proposed model is. The fact that only the AP value of Query 12 out of 20 randomly selected queries is below 0.90 and the average mAP value calculated for all queries is approximately 0.9538, which is higher than the individual models, shows that the Ensemble approach offers high accuracy and effective retrieval success in most cases. When looking at the PR curves, it is observed that most queries maintain high precision values in a wide recall range and exhibit a very stable performance. This is an indication that the feature-level fusion approach, which successfully integrates the complementary features of different deep learning architectures, and the cosine similarity used as the similarity measure, works effectively.

The fact that the cosine similarity scores obtained in the result returned by the model for the sample query image reach almost 1.0 reveals that the system succeeds in positioning similar images in the same vector space and thus can rank the most similar images to the query image from the dataset with high accuracy.

In this study, performance metrics such as classification accuracy and mean Average Precision (mAP) were reported based on a single, fixed train/test split. Due to this experimental setup, traditional statistical significance testing (e.g., McNemar’s test or paired *t*-tests) was not performed, as such methods require multiple independent trials or resampling to yield meaningful variance estimates. Because the Kaggle Melanoma Cancer Image dataset has a fixed structure with a specific training/test split, we maintained the same layout for comparison with the previous literature. Therefore, implementing k-fold cross-validation was technically not feasible. However, to compensate for this shortcoming, our model’s performance is reported in detail using other metrics such as accuracy, precision, recall, F1-score (Table 2 and Table 3), ROC curves, confusion matrix, and mAP (Table 4 and Table 5). All of these metrics provide comprehensive insights into the model’s performance in both classification and content-based image retrieval. Additionally, to statistically demonstrate the reliability of the performance metrics in the test data, confidence intervals were calculated using bootstrap sampling on the obtained test results. Therefore, the 95% confidence interval (CI) was calculated based on the bootstrap sampling value (*n* = 100 iterations) using Equation (7), based on the accuracy and mAP metrics. The constant 1.96 represents the z-score for the 95% confidence level. *x* is the mean value, and *s* indicates the standard deviation. Table 6 shows the variances of the accuracy performance of different models according to varying std values using Bootstrap sampling.

(7) $${CI}_{95\%}=\bar{x}\pm 1.96\times \frac{s}{\sqrt{n}}$$

According to Table 6, the classification accuracy of the Ensemble model was 95.25% (95% CI: 95.18–95.32%), indicating high consistency across bootstrap samples. In contrast, the ViT model showed more variability with an accuracy of 88.25% (95% CI: 88.10–88.40%). Similarly, using Equation (7), 95% confidence intervals were calculated for the mAP values obtained by the different models, and the results are shown in Table 7.

According to Table 7, the mAP of the proposed Ensemble system was 0.9538 with a narrow 95% confidence interval (CI: 0.9532–0.9544), indicating high consistency in retrieval performance. In comparison, the ViT model yielded a lower and more variable mAP of 0.7539 (95% CI: 0.7521–0.7557), reflecting greater instability in precision across bootstrap samples.

Recent advancements in hybrid and prompt-based AI models have introduced novel opportunities for improving performance in data-scarce medical image analysis tasks. For instance, the MHKD framework demonstrates how multi-step hybrid knowledge distillation can be leveraged to maintain diagnostic accuracy even in low-resolution whole-slide images, which is highly relevant to practical clinical settings with suboptimal image quality [43]. Similarly, the work by Zhang et al. on vision–language models shows that combining image features with semantic prompts can significantly enhance nuclei segmentation and classification, indicating the potential of cross-modal learning in histopathological contexts [44]. Furthermore, the low-shot learning strategy explored in [45] opens new avenues for reducing dependency on large annotated datasets. Integrating such prompt-based pre-training into melanoma classification systems could facilitate better generalization and performance in low-resource scenarios, particularly when dealing with rare lesion subtypes. These methodologies suggest promising directions for future extensions of our approach, especially in enhancing the robustness and scalability of ensemble-based classification and CBIR systems in dermatological applications.

In this study, we utilized only the publicly available “Melanoma Cancer Image” dataset from Kaggle to develop and evaluate our model. While this dataset enabled us to achieve high classification accuracy, it may not adequately capture real-world diversity in terms of skin tone variation, lighting conditions, imaging artifacts, and device-specific differences. As a future direction, we plan to perform external validation using dermoscopic images collected from the ISIC (International Skin Imaging Collaboration) archive and multiple clinical centers. This will allow us to further assess the generalizability and robustness of the proposed model in more heterogeneous and realistic environments. Although our ensemble and CBIR results are encouraging on the publicly available Kaggle melanoma dataset, they are derived from a single-center, retrospective dataset with standardized imaging conditions. To ensure generalizability and clinical relevance, external validation on heterogeneous datasets capturing variability in dermatoscopic devices, patient skin types, and real-world imaging artifacts is necessary [46].

In practice, our dual-task ensemble could be deployed as a plugin for existing dermoscopy workstations, streaming live dermoscopic video to provide both an on-screen malignancy probability and, alongside each capture, a ranked display of visually similar past cases [47]. Such real-time decision support could accelerate diagnosis and boost confidence, especially for less experienced clinicians. Looking ahead, prospective, multicenter validation studies directly comparing our system’s predictions and retrievals against board-certified dermatologists, as well as integration trials within electronic health record environments to fully assess workflow impact and patient outcomes, could be conducted.

It is important to note that the proposed Ensemble-based model is designed as a diagnostic support system for melanoma detection rather than an all-encompassing solution ready for real-world deployment. Consequently, this study does not evaluate system-level performance aspects such as inference time and memory consumption. While the ensemble fusion of multiple models improves classification and retrieval accuracy, it also introduces architectural complexity that may not be optimal for real-time applications. In future work, we plan to address these limitations by applying model compression techniques such as pruning and quantization, along with deployment optimizations through TensorRT or ONNX. These steps will help reduce computational load and enhance the feasibility of the proposed system in practical clinical environments.

## 5. Conclusions

In this study, in order to classify melanoma images and increase the performance of CBIR systems, classification was performed with different TL-based architectures such as DenseNet121, InceptionV3, ViT, and Xception. Ensemble learning was performed using these models, and then a hybrid model, the multi-model (feature-level fusion), was developed in which the deep features obtained from these models were integrated with the fusion method at the feature level. The Ensemble model, in which individual models were weighted, provided high classification accuracy, and the multi-model (feature-level fusion) model, which was developed to increase CBIR performance, provided a significant improvement in CBIR operations by highlighting the strengths offered by individual models thanks to the optimized layers and horizontal merging of feature vectors. According to the obtained results, the Ensemble model achieved a 95.25% accuracy rate on the test dataset, which showed a significant increase in comparisons made with other TL-based models. At the same time, the mAP value calculated from 20 randomly selected queries with the multi-model was calculated as 0.9538. This situation revealed the ability to rank similar images with almost 100% cosine similarity scores in the CBIR process. The high and stable precision values in the PR curves and the consistency in wide recall intervals prove that the model can effectively blend the semantic representations of the images. This study shows that the Ensemble method and the multi-model developed for CBIR have great potential in terms of early diagnosis, accurate classification, and fast similarity matching in clinical applications such as melanoma detection and image retrieval; it will also constitute an important reference and resource for future studies.

## Figures and Tables

**Table 1:** Two-label confusion matrix.

		Predicted	
		Positive	Negative
Actual	Positive	TP	FP
	Negative	FN	TN

**Table 2:** Performance results obtained from models using Transfer learning.

Models	Accuracy	Precision	Recall	F1-Score
DenseNet121	94.50%	93.90%	95.04%	94.47%
InceptionV3	91.20%	93.20%	89.62%	91.37%
ViT	88.25%	90.50%	86.60%	88.50%
Xception	93.80%	93.00%	94.51%	93.75%

**Table 3:** Performance results obtained from Ensemble learning model.

Model	Accuracy	Precision	Recall	F1-Score
Ensemble learning	95.25%	94.20%	96.22%	95.20%

**Table 5:** CBIR performance results of the multi-model approach for the mAP values of 20 queries.

Models	Mean Average Precision (mAP)
Multi-model fusion	0.9538

**Table 6:** Accuracy results of models—95% CI (*n* = 100).

Models	Accuracy (%)	Std (%)	CI Width (±)	95% CI Range
DenseNet121	94.50%	0.45	±0.088	(94.41–94.59)
InceptionV3	91.20%	0.65	±0.127	(91.07–91.33)
ViT	88.25%	0.75	±0.147	(88.10–88.40)
Xception	93.80%	0.50	±0.098	(93.70–93.90)
Ensemble Learning	95.25%	0.35	±0.069	(95.18–95.32)

**Table 7:** The mAP results of models—95% *CI* (*n* = 100).

Models	mAP (%)	Std (%)	CI Width (±)	95% CI Range
DenseNet121	0.9496	0.0040	±0.000784	(0.9488–0.9504)
InceptionV3	0.7922	0.0070	±0.001372	(0.7908–0.7936)
ViT	0.7539	0.0090	±0.001764	(0.7521–0.7557)
Xception	0.9171	0.0050	±0.000980	(0.9161–0.9181)
Multi-model fusion	0.9538	0.0030	±0.000588	(0.9532–0.9544)

## Data Availability

All relevant data are fully available within the manuscript without restriction. The Kaggle data that support this study’s conclusions are publicly available online: https://www.kaggle.com/datasets/bhaveshmittal/melanoma-cancer-dataset (accessed on 28 May 2025).
